# Supplementary material for: Preconception Diet Interventions in Obese Outbred Mice and the Impact on Female Offspring Metabolic Health and Oocyte Quality
Source: Int J Mol Sci. 2024 Feb 13;25(4):2236. doi: 10.3390/ijms25042236 (PMC10888670; doi:10.3390/ijms25042236)
Supplement: Supplementary file 1 [file ijms-25-02236-s001.zip › ijms-2820800-supplementary.pdf]

Supplementary data

Preconception Diet Interventions in Obese Outbred Mice and the Impact on Female Offspring Metabolic Health and Oocyte Quality

Ben Meulders [1], Waleed F. A. Marei [1,2], Inne Xhonneux [1], Lien Loier [1], Anouk Smits [1] and Jo L. M. R. Leroy [1,*]

[1] Gamete Research Centre, Laboratory of Veterinary Physiology and Biochemistry, Department of Veterinary Sciences, University of Antwerp, 2610 Antwerp, Belgium
[2] Faculty of Veterinary Medicine, Department of Theriogenology, Cairo University, 12211 Giza, Egypt
* Correspondence: jo.leroy@uantwerp.be (J.L.M.R.L.)

Supplementary Figure S1: Serum total cholesterol (A), glucose (B), triglyceride (C), and non-esterified fatty acid (NEFA; D) concentrations in female offspring born to HF/HS = high-fat/high-sugar diet, DN = diet normalization, CR = caloric restriction, or CONT = control diet. Each bar shows mean +/- SEM.

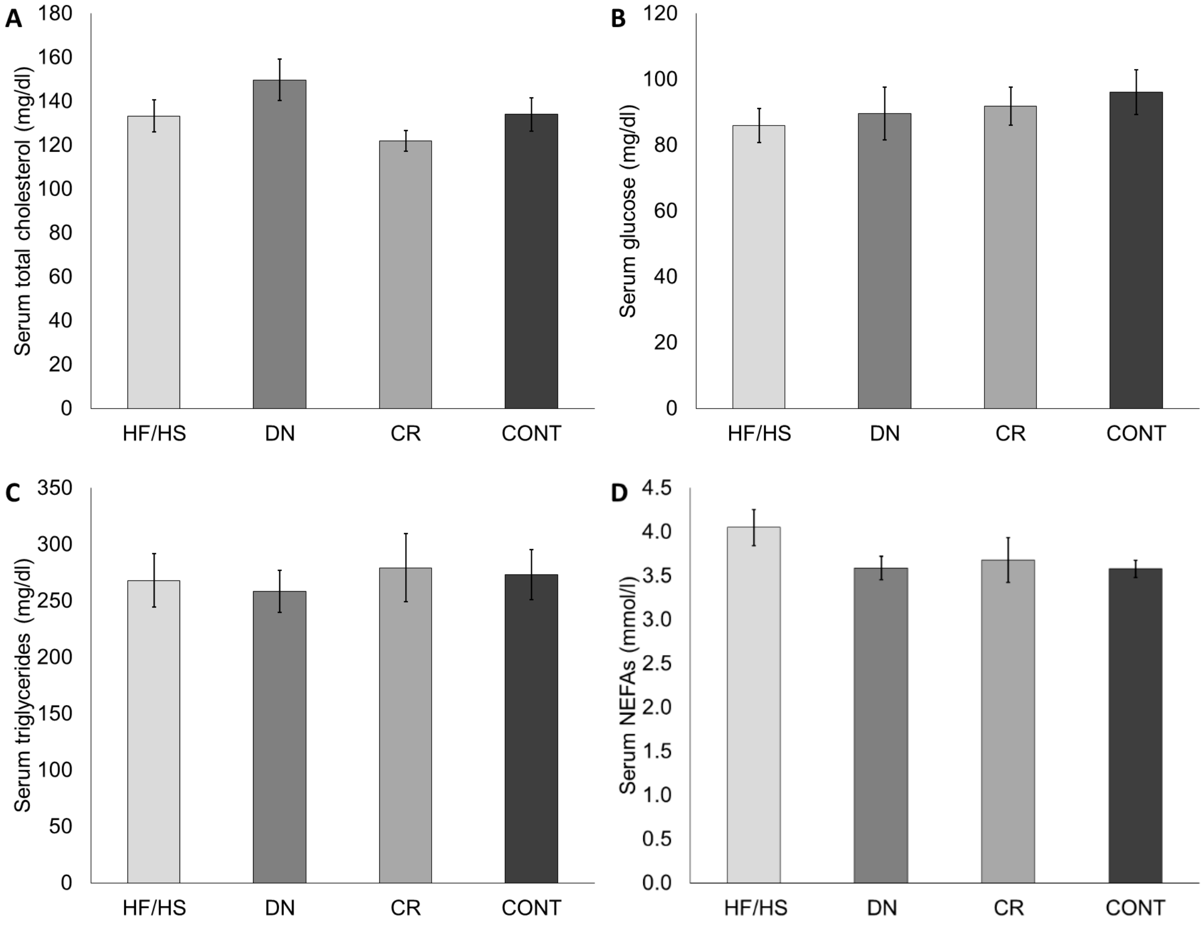

Supplementary Figure S2: Classification of mitochondrial ultrastructure in transmission electron microscope (TEM) images of F1 oocytes (16500x magnification). Mitochondria were classified as described by Marei et al. (2020). Firstly, mitochondria with a spherical homogeneous shape (A) or regular vacuoles (B) were classified as normal. On the other hand, mitochondria were classified as abnormal if they showed a loose inner membrane (spherical (C) or non-spherical (D)), dumbbell shape (E), elongation (F), degeneration (G), rosepetal appearance (H) or increased electron density (I).

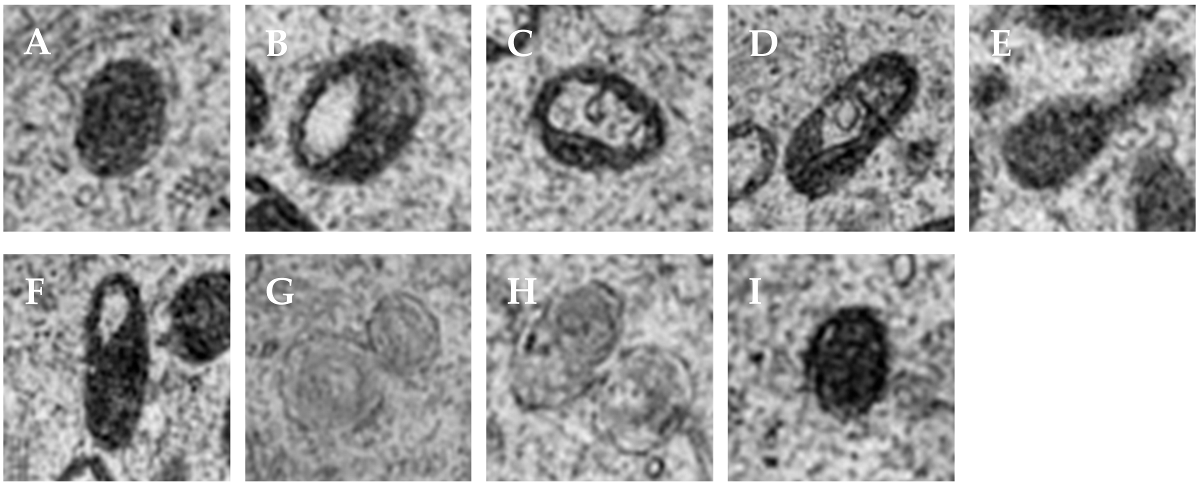

Supplementary Figure S3: Representative confocal images of central z-plane from F1 oocytes after immunostaining for global DNA methylation (5mC). Oocytes were examined under an SP8 confocal microscope (Leica, Diegem, Belgium) and equipped with a white laser source (WLL, Leica) at excitation/emission 488/525 nm (to visualize FITC-labelled 5mC). The nucleus (full arrow) is indicated in the figures. The demarcation of the oocyte is indicated with a dotted line.

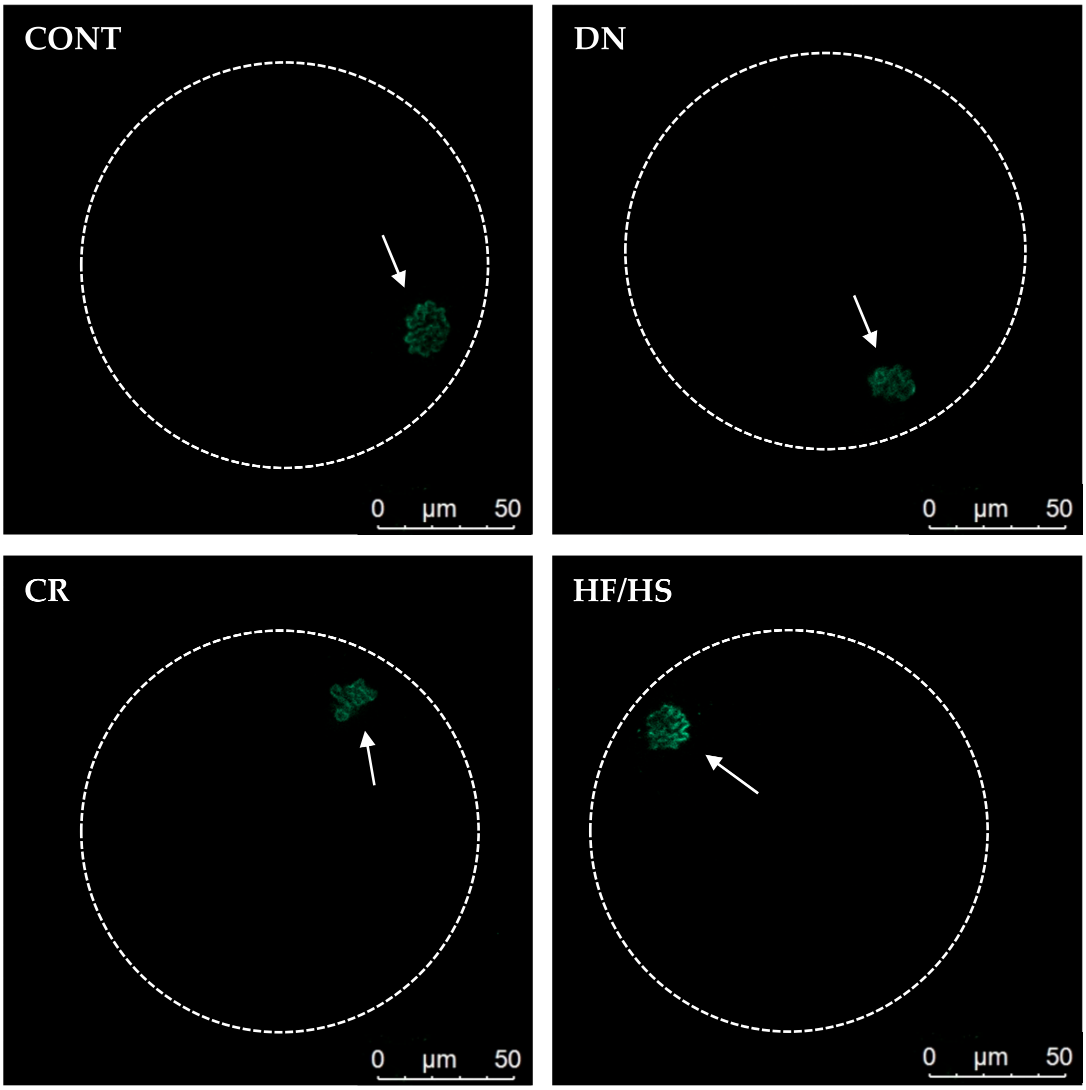

Supplementary Table S1: Overview of the 17 Differentially Regulated Proteins (DRPs) that were observed in the cluster which contained a pattern where these proteins were downregulated in the HF/HS group compared to CONT, the DN group was similar to CONT, while in CR some proteins were also downregulated compared to CONT. Here, for each of these DRPs the protein name, name of the corresponding gene, and Gene Ontology (GO) biological processes in which these genes are enriched are listed.

Protein name	Gene name	Biological Process (GO)
Stathmin 1	Stmn1	GO:0070494 regulation of thrombin-activated receptor signaling pathway<br>GO:0070495 negative regulation of thrombin-activated receptor signaling pathway<br>GO:0048012 hepatocyte growth factor receptor signaling pathway
Myosin, heavy polypeptide 6, cardiac muscle, alpha	Myh6	GO:0007522 visceral muscle development<br>GO:0030049 muscle filament sliding<br>GO:0055009 atrial cardiac muscle tissue morphogenesis
Myosin, heavy polypeptide 4, skeletal muscle	Myh4	GO:0014823 response to activity<br>GO:0006936 muscle contraction<br>GO:0003012 muscle system process
Guanine nucleotide binding protein (G protein), beta 1	Gnb1	GO:0007603 phototransduction, visible light<br>GO:0010659 cardiac muscle cell apoptotic process<br>GO:0010658 striated muscle cell apoptotic process
Proteasome (prosome, macropain) inhibitor subunit 1	Psmf1	GO:1901799 negative regulation of proteasomal protein catabolic process<br>GO:1903051 negative regulation of proteolysis involved in protein catabolic process<br>GO:0042177 negative regulation of protein catabolic process
Protein disulfide isomerase associated 3	Pdia3	GO:1903334 positive regulation of protein folding<br>GO:0002397 MHC class I protein complex assembly<br>GO:0002502 peptide antigen assembly with MHC class I protein complex
N(alpha)-acetyltransferase 50, NatE catalytic subunit	Naa50	GO:0034087 establishment of mitotic sister chromatid cohesion<br>GO:0071962 mitotic sister chromatid cohesion, centromeric<br>GO:0034085 establishment of sister chromatid cohesion
Hypoxia up-regulated 1	Hyou1	GO:1903382 negative regulation of endoplasmic reticulum stress-induced neuron intrinsic apoptotic signaling pathway<br>GO:1903381 regulation of endoplasmic reticulum stress-induced neuron intrinsic apoptotic signaling pathway<br>GO:1903298 negative regulation of hypoxia-induced intrinsic apoptotic signaling pathway
Adenylate kinase 4	Ak4	GO:0009188 ribonucleoside diphosphate biosynthetic process<br>GO:0009133 nucleoside diphosphate biosynthetic process<br>GO:0009185 ribonucleoside diphosphate metabolic process
Mitochondrial ribosomal protein S36	Mrps36	GO:0006103 2-oxoglutarate metabolic process<br>GO:0006099 tricarboxylic acid cycle<br>GO:0043648 dicarboxylic acid metabolic process
Tropomyosin 3, gamma	Tpm3	GO:0006936 muscle contraction<br>GO:0003012 muscle system process<br>GO:0007015 actin filament organization
Tropomyosin 3, related sequence 7	Tpm3-rs7	GO:0006936 muscle contraction<br>GO:0003012 muscle system process<br>GO:0007015 actin filament organization

Ubiquitin carboxy-terminal hydrolase L1	Uchl1	GO:0007412 axon target recognition<br>GO:0002176 male germ cell proliferation<br>GO:0036093 germ cell proliferation
Tropomyosin 1, alpha	Tpm1	GO:0003065 positive regulation of heart rate by epinephrine<br>GO:0001996 positive regulation of heart rate by epinephrine-norepinephrine<br>GO:0003062 regulation of heart rate by chemical signal
ATP synthase inhibitory factor subunit 1	Atpif1	GO:0051882 mitochondrial depolarization<br>GO:1901030 positive regulation of mitochondrial outer membrane permeabilization involved in apoptotic signaling pathway<br>GO:1904925 positive regulation of autophagy of mitochondrion in response to mitochondrial depolarization
Myotubularin related protein 14	Mtmr14	GO:0016311 dephosphorylation<br>GO:0006796 phosphate-containing compound metabolic process<br>GO:0006793 phosphorus metabolic process
Cytochrome c oxidase subunit 5A	Cox5a	GO:0006123 mitochondrial electron transport, cytochrome c to oxygen<br>GO:0019646 aerobic electron transport chain<br>GO:0042775 mitochondrial ATP synthesis coupled electron transport
Eukaryotic translation initiation factor 2, subunit 3, structural gene X-linked	Eif2s3x	GO:0001731 formation of translation preinitiation complex<br>GO:0002183 cytoplasmic translational initiation<br>GO:0006413 translational initiation
Eukaryotic translation initiation factor 2, subunit 3, structural gene Y-linked	Eif2s3y	GO:0001731 formation of translation preinitiation complex<br>GO:0002183 cytoplasmic translational initiation<br>GO:0006413 translational initiation
